# Reaction Route Selection for Cellulose Hydrogenolysis into C_2_/C_3_ Glycols by ZnO-Modified Ni-W/β-zeolite Catalysts

**DOI:** 10.1038/s41598-019-48103-6

**Published:** 2019-08-16

**Authors:** Minyan Gu, Zheng Shen, Long Yang, Wenjie Dong, Ling Kong, Wei Zhang, Bo-Yu Peng, Yalei Zhang

**Affiliations:** 1 State Key Laboratory of Pollution Control and Resources Reuse, Key Laboratory of Yangtze River Water Environment of MOE, National Engineering Research Center of Protected Agriculture, Shanghai Engineering Research Center of Protected Agriculture, Tongji University, Shanghai, 200092 China; 2 College of Quality and Safety Engineering, China Jiliang University, Hangzhou, 310018 China; 3 Shanghai Institute of Pollution Control and Ecological Security, Shanghai, 200092 China

**Keywords:** Chemistry, Catalysis, Chemical engineering, Green chemistry

## Abstract

A β-zeolite-supported nickel and tungsten catalyst (Ni-W/β) was employed to generate C_2_/C_3_ glycols (ethylene and propylene glycols) in a satisfactory yield from cellulose. After optimizing the acidity of the support, the Ni-W synergy and the co-catalyst, the yield of C_2_/C_3_ glycols reached 70.1% (C %), with propylene glycol accounting for 51.1% of the product. This performance was attributed to the effective control of the major reaction steps, namely, hydrolysis, isomerization, retro-aldol condensation and hydrogenation, by the tailored Ni-W-ZnO/β catalyst. The characterization and reaction results indicated that the cellulose hydrolysis step was promoted by the appropriate acidic sites of the β-zeolite, and the reaction routes to C_2_/C_3_ glycols were influenced by the mass loading of Ni-W through the synergy of nickel and tungsten oxide, in which Ni is effective in the hydrogenation while W facilitates bond cleavage via a retro-aldol condensation (C_6_ to C_2_/C_3_). Moreover, with the leaching of metal during four cycles of reuse, the catalytic performance was also influenced by the synergy of Ni and W. In addition, the isomerization of glucose to fructose was promoted by ZnO and afforded a high yield of propylene glycol.

## Introduction

With diminishing fossil resources and increasing environmental concerns worldwide, searching for alternative fuels has attracted great interest in recent years. As a vital renewable alternative to fossil fuels, cellulose is the most abundant source of biomass and has received considerable attention. Consequently, different conversion routes for cellulose have been explored to achieve high energy efficiency and atom economy^1–3^. Cellulose has been selectively converted into different chemicals, such as oligomers^3^, glucose^2^, HMF^1^, sorbitol^4^, hexitols^5^ and C_2_/C_3_ glycols (ethylene glycol and propylene glycol, EG/PG) through a series of chemical reactions, including hydrolysis, dehydration and hydrogenolysis. Among them, the hydrogenolysis of cellulose to C_2_/C_3_ glycols is particularly noteworthy because of the versatile applications of glycols directly and as platform molecules in the synthesis of fuels and value-added products, including polyesters and antifreeze^6^.

The catalytic conversion of cellulose is a complex reaction network comprising hydrolysis, retro-aldol condensation, hydrogenation, isomerization, dehydrogenation, thermal side reactions, etc^7^. In addition to C_2_/C_3_ glycols, a variety of by-products, such as sorbitol, mannitol, xylitol, 1,2-butanediol, and glycerol, can be coproduced^7^. Based on previous studies, three reaction steps are critical for cellulose conversion to EG: hydrolysis, retro-aldol condensation, and hydrogenation. For PG, the isomerization of glucose is also involved. To enhance the above reaction steps and increase the yield of C_2_/C_3_ glycols, a series of effective metals or metallic oxides, such as tungsten derivatives^8–13^, tin derivatives^14,15^, lanthanides^6^, niobium^16^ and zinc^17^, were employed in the catalytic system. Deng *et al*. modified Pt/Al_2_O_3_^14^ and Ni/Al_2_O_3_^18^ catalysts by SnO_x_ to enhance the conversion of cellulose to C_2_/C_3_ glycols. Using these two catalysts, carbon selectivity of C_6_ products decreased from 43.3 to 0.8% and 63.3 to 0.2%, respectively, which indicated that SnO_x_ played a significant role in C-C bond cleavage via retro-aldol condensations. In addition, the carbon selectivity for C_3_ products increased from 13.5 to 28.6% and 12.3 to 56.9%, respectively, demonstrating the advantages of SnO_x_ in glucose-fructose isomerization. However, the cellulose conversions obtained with SnO_x_-modified Pt/Al_2_O_3_ and Ni/Al_2_O_3_ catalysts remained below 23% (C%), which was attributed to the fact that these catalysts cannot facilitate cellulose hydrolysis. Sun *et al*. designed Sn powder- and SnO-modified Ni/AC catalysts to enhance the retro-aldol condensation step in cellulose hydrogenolysis. The carbon yield of EG obtained with the Sn powder modification reached 57.6%, and the carbon yield of PG/EG with the SnO-modified catalyst reached 22.9%/32.2%. Our previous studies^19,20^ showed results similar to those obtained in the Sn-enhanced retro-aldol condensation step. In addition to Sn species, W species also facilitated cellulose or glucose hydrogenolysis into C_2_/C_3_ glycols. WO_x_ was introduced onto a Pd/Al_2_O_3_ catalyst by Liu *et al*.^21^ to increase the yield of PG from glucose. The results suggested that WO_x_ enhanced glucose-fructose isomerization and achieved PG yields up to 56.1% (C%). They^22^ also introduced WO_x_ into a Cu/Al_2_O_3_ catalyst and achieved a PG carbon yield of 38.1%, which was attributed to the glucose-fructose isomerization and retro-aldol condensation steps. In addition, W_2_C/AC, Ni-WP/AC, Ru/C-WO_3_ + C_act_, Ru/AC-H_2_WO_4_ and Cu/CuCr_2_O_3_ provided C_2_/C_3_ glycol carbon yields of 32.8%^8^, 52.4%^9^, 12.8%^11^, 62.0%^12^ and 43.9%^13^, respectively.

The above studies realized the increase of C_2_/C_3_ glycols yields by enhancing the isomerization or retro-aldol condensation steps. Focusing on the synergy of the hydrolysis, hydrogenation, retro-aldol condensation and hydrogenation steps in C_2_/C_3_ generation is more important than focusing on a single step. In view of this, we will fully consider cellulose hydrolysis, hydrogenation and the synergy of the hydrogenation and retro-aldol condensation in this paper. In order to increase the C_2_/C_3_ generation, the reaction process was expected as below: when cellulose hydrolysis took place, hydrogenation was not expected to occur because the products of the hydrogenation reaction (C_6_ alcohols) were much more stable and more difficult to convert to C_2_/C_3_ species than C_6_ sugars. Then, after the C-C bond cleavage in the sugars via a retro-aldol condensation (C_6_ to C_2_/C_3_), hydrogenation occurs immediately to generate C_2_/C_3_ glycols, avoiding the occurrence of other side reactions. When the reaction reached this point, further C-C bond cleavage should not occur. Otherwise, smaller molecules could be generated from the C_2_/C_3_ glycols. Furthermore, as a more valuable product, PG is more difficult to produce because the cleavage of the C_3_-C_3_ bond in C_6_ sugars should occur, not C_2_–C_4_.

Herein, to balance the hydrolysis, retro-aldol condensation and hydrogenation and to realize the selective conversion of cellulose into C_2_/C_3_ glycols, three aspects will be investigated and discussed: 1) a tailored catalyst will be designed and synthesized, including support selection, support structure modification, control of the Ni-W synergy and co-catalyst screening; 2) the effect of the above factors on the reaction route and the overall mechanism will be studied via a series of catalytic reactions and analyses; 3) the reuse performance and the relationship between Ni-W leaching and product distribution during reuse will be utilized to verify the effect of Ni-W on reaction route selection.

## Results and Discussion

### The effect of the acidity of the β-zeolite

**Figure 1:**
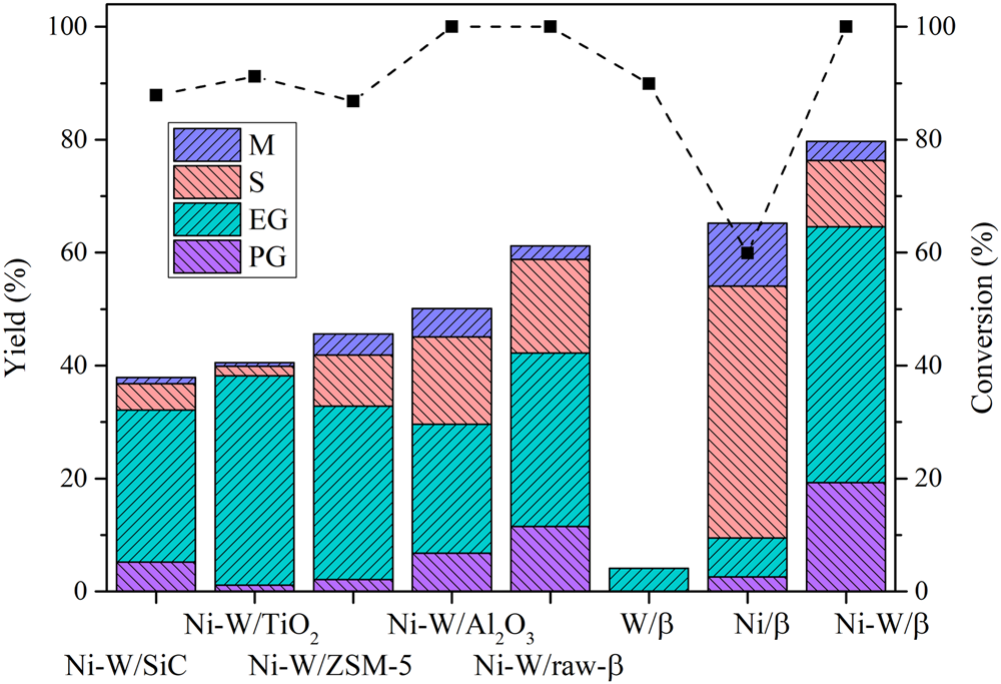
Catalytic reactions on different supports. Reaction conditions: 30 min, 6 MPa H_2_, 245 °C, 50 mL of H_2_O, 0.5 g of cellulose, 0.15 g of catalyst. EG: ethylene glycol; PG: 1,2-propylene glycol; S: sorbitol; and M: mannitol. Other products not listed include methane, methanol, ethanol, 1-propanol, glycerol, 1,2-butanediol, erythritol, 1,2-pentanediol, 1,2-hexanediol, furfural, furan and other unknown products.

Because of the obvious importance of the catalytic support, which not only physically supports and improves the dispersion of the metal but also provides acid sites, different supports (Al_2_O_3_, TiO_2_, SiC, ZSM-5, and raw β-zeolite) were screened to determine the optimal combination for cellulose and glycols based on Ni-W bimetallic synergy. As displayed in Fig. 1, mannitol (M), sorbitol (S), EG and PG (C_2_/C_3_ glycols) as the major alcohol products were exhibited. There was a significant difference between the total yield of M, S, PG and EG, the yields of C_2_/C_3_ glycols, and the conversions obtained with Ni-W/SiC, Ni-W/TiO_2_, Ni-W/ZSM-5, Ni-W/Al_2_O_3_ and Ni-W/raw-β catalysts. Ni-W/raw-β exhibited the highest reactivity. To understand the reason for the superiority of Ni-W/raw-β, the physicochemical properties of the catalysts were investigated, as displayed in Table S1 (entries 1 to 6). The total loading of Ni and W remained the same for each case, whereas BET surface area and acidity varied considerably between catalysts. The BET surface areas of Ni-W/SiC, Ni-W/TiO_2_, Ni-W/ZSM-5, Ni-W/Al_2_O_3_ and Ni-W/raw-β were 56, 42, 410, 134, 317 m^2^/g, and the corresponding yields of C_2_/C_3_ glycols were 32.1, 38.2, 32.8, 29.6 and 42.2%, respectively. The total yields of the major alcohol products were 37.9, 40.5, 45.6, 50.1 and 61.2%, respectively. The results suggested that there was no obvious correlation between the BET surface area and the reactivities on different supports, indicating that the surface area was not the primary cause of the superiority of Ni-W/raw-β^23^. The NH_3_-TPD curves (Figure S1(a)) showed a large difference in the acidity of the catalysts. The raw-β-supported Ni-W catalyst exhibited a significantly larger peak area than the other supports. The desorption peaks of Ni-W/raw-β centred at approximately 200 °C and 450 °C indicated that it had weak and medium-strength acid sites. In contrast, fewer acid sites were seen with other catalysts, such as Ni-W/Al_2_O_3_, which had some weak and medium-strength sites, Ni-W/TiO_2_, which had few weak and strong sites, and Ni-W/SiC, which had no obvious desorption peaks. The quantitative acidity data in Table S1 (entries 1 to 6) further illustrated that Ni-W/raw-β exhibited significantly more acid sites. As Table S1 (entries 1 to 6) shows, the acidity of Ni-W/SiC were below the limit suitable for this test and the acidity of Ni-W/TiO_2_, Ni-W/ZSM-5, Ni-W/Al_2_O_3_ and Ni-W/raw-β were 0.15, 0.17, 0.62 and 1.71 mmol/g, respectively, and the total yields of C_2_/C_3_ glycols, mannitol and sorbitol were 37.9%, 40.5%, 45.6%, 50.1% and 61.2%, respectively. The relationship between the acidity and the total yield of C_2_/C_3_ glycols, mannitol and sorbitol further supported that the acidity was an important factor in the reaction performance. The effect of acidity on performance is discussed in the mechanism section. The mechanism of the hydrolysis of cellulose into glucose might be promoted by the abundant acid sites in β-zeolite. This was also demonstrated by Lazaridis, P. A., *et al*.^24^ who showed that cellulose can be degraded into glucose and hexitols through conventional acidic hydrolysis and then converted into C_2_–C_6_ alcohols.

To optimize the structure and performance of Ni-W/raw-β, a series of modifications were tested, and of these, removal of the aluminium (denoted Ni-W/β) provided remarkable results. As displayed in Fig. 1, the yield of C_2_/C_3_ glycols obtained with Ni-W/β increased from 42.2% to 64.6% upon removal of the aluminium. In this process, the aluminium was removed from the β zeolite by the method mentioned in our previous studies^25^ to generate empty sites for the loading of other metals (e.g., Ni and W) and redistribution of the acid sites in the catalysts. As Figure S1(a) shows, the ammonia desorption peaks of Ni-W/β were shifted from 200 and 450 to 650 °C, indicating that the strength of the acid sites was enhanced by dealumination, although the quantity of sites did not increase. This result suggested that not only the quantity of acid sites but also the strength of the acid sites would influence the performance of Ni-W/β. As Ni-W/β was superior in the hydrogenolysis of cellulose, dealuminated β-zeolite was chosen as the support for the Ni-W catalyst in the following experiments.

### The synergistic effect of Ni and W

**Figure 2:**
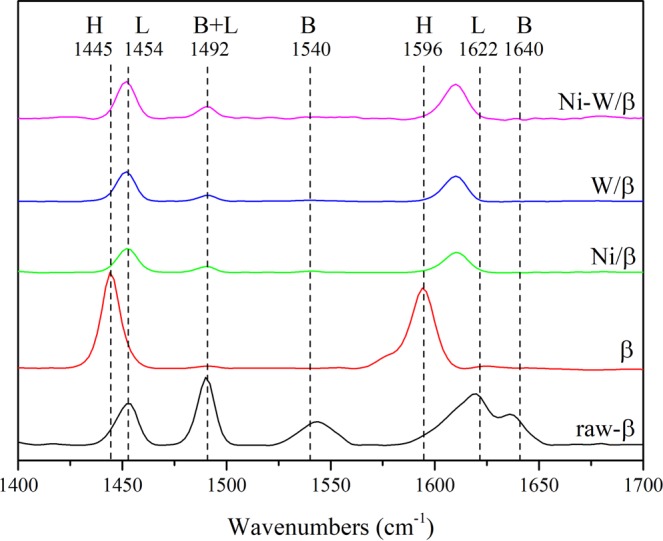
Py-IR profile of β-based catalysts adsorbed at 150 °C.

As mentioned above, the acid sites played an important role in the production of glycols, especially in the acidic hydrolysis of cellulose^20,25^. Therefore, we firstly investigated the synergistic effects of Ni and W on the acid sites. As shown in Fig. 2, although both the Brønsted acidity (1540 cm^−1^) and Lewis acidity (1492 cm^−1^) of raw β-zeolite were removed during the dealumination by HNO_3_ solution, Ni-W/β still showed higher glycol yields than the other catalysts (Fig. 1), which may be attributed to the function of the Ni and W species in the acid sites. The assumption is confirmed by the data in Fig. 2; after the loading of Ni and W, great changes occurred in the acidity of the catalyst, suggesting that Ni provided abundant Lewis acid sites in the β-zeolite and that W provided both Brønsted and Lewis acid sites, which could ensure the success of cellulose hydrolysis and hydrogenolysis. Moreover, Fig. 1 shows that Ni-W/β exhibited high catalytic activity, but Ni/β and W/β both exhibited low activity, which indicated that Ni-W bimetallic synergy, and not the individual effects of Ni or W, facilitated the C_2_/C_3_ glycols production. In order to more clearly explain the synergy of Ni and W on acidity, the NH_3_-TPD experiment was showed in Figure S1(b). Similar to Py-IR result, after dealumination, β lost almost all of its acidity. The only small peaks near 150 °C might attributed to the OH groups formed by alumina vacancy, which was similar to the H peaks in Py-IR. After the loading of Ni or W on β (deAl), the catalytic acidity was significantly increased. While Ni and W loaded on β together, the acidity of catalyst increased more significantly. As shown in Table S1 (entries 6 to 10), the synergy was clearly observed that the acidity of Ni-W/β (1.67 mmol/g) was stronger than the sum of Ni/β and W/β (1.20 mmol/g).

**Figure 3:**
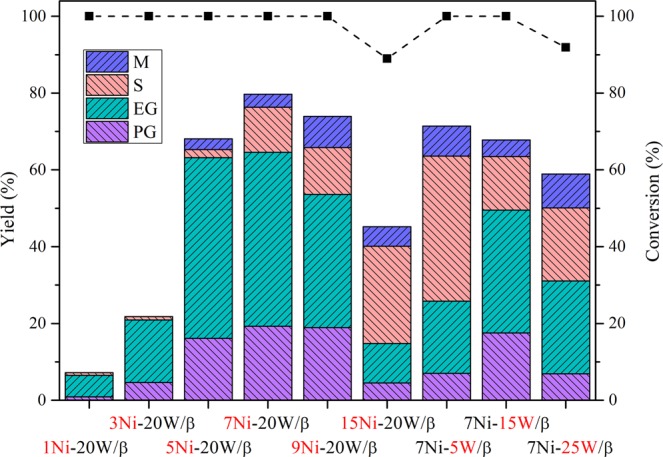
Catalytic reaction performance over the Ni-W/β catalysts with various Ni-W loading. Reaction conditions: 30 min, 6 MPa H_2_, 245 °C, 50 mL of H_2_O, 0.5 g of cellulose, 0.15 g of catalyst. EG: ethylene glycol; PG: 1,2-propylene glycol; S: sorbitol; M: and mannitol.

To further probe the role of Ni-W in the reaction, different loading of Ni and W were employed in the Ni-W/β catalysts. As displayed in Fig. 3, the C_2_/C_3_ glycols yield increased from 6.5% to 64.6% when the loading of W remained at 20 wt % and the weight percentage of Ni increased from 1 wt % to 7 wt %. This increase may be attributed to nickel, as the metal responsible for hydrogenation, providing more hydrogenation sites when a greater amount was used, which also increased the yield of hydrogenation products, sorbitol and mannitol^8^. With a further increase in the Ni weight percentage from 7 to 15 wt %, the C_2_/C_3_ glycols decreased from 64.6 to 14.8% and the cellulose conversion decreased from 100.0 to 89.0%. These results might be a result of the facile aggregation of the excess nickel, shielding the mesopores and W sites and the decrease of catalytic acidity, which could be verified by the BET (Table S2, Entries 6 to 11), TEM (Figure S2), XRD results (Figure S3d) and NH_3_-TPD results (Table S3). Table S2 (Entries 6 to 11) shows that the BET surface area, as well as the pore volume, decreased with increasing Ni weight percentage. As shown in Figure S2, undesired particle aggregation was more apparent on 15Ni-20W/β than it was on 1Ni-, 5Ni-, 7Ni- and 9Ni-20W/β. Furthermore, the poor performance when excess Ni is present in catalytic structure is explained in Figure S3(d), as significant NiWO_4_ diffraction peaks were observed, suggesting that the excess Ni promoted the formation of NiWO_4_, decreasing the number of active Ni and W particles. As shown in Table S3, the acidity of 15Ni-20W/β was lower than that of other catalysts, which might attributed to the generation of NiWO_4_ when Ni was excess and the generated NiWO_4_ could not provide acidity sites. The above evidence all confirm that excess nickel has a negative effect on the hydrogenolysis of cellulose into C_2_/C_3_ glycols.

When the loading of Ni remained at 7 wt % and the weight percentage of W increased from 5 wt % to 25 wt %, the maximum C_2_/C_3_ glycols yield was obtained by 7Ni-20W/β (Fig. 3), indicating that the appropriate amounts of W and Ni significantly influenced the C_2_/C_3_ glycols production. As shown in Fig. 3, the C_2_/C_3_ glycols yield obtained with 7Ni-5W/β was only 25.8%, while the yield of sorbitol and mannitol was up to 44.6%. The formation of a large amount of C_6_ sugar alcohols suggested that the insufficient quantity of W species (5 wt %) impeded the bond cleavage from C_6_ to C_2_/C_3_ products based on the understood mechanism that glycols were formed by the hydrolysis of cellulose into glucose and subsequent bond cleavage of glucose/fructose^7,11,26^ via a retro-aldol condensation (RAC). Therefore, as Fig. 3 shows, with increasing W content from 5 to 20 wt %, the yield of C_6_ products decreased from 44.6 to 10.3%, and the yield of C_2_/C_3_ glycols increased from 25.8 to 64.6%, which further confirmed that the W species play an important role in bond cleavage in the C_6_ species. However, an especially low yield of glycols was obtained with 7Ni-25W/β, which could be explained by the formation of NiWO_4_ (Figure S3), the small BET surface area (Table S2) and the lower acidity (Table S3).

We could conclude from the above results that excessive amounts of either Ni or W negatively impacted the performance of the catalyst. The cooperation of Ni and W active sites was required for this reaction, and C_2_/C_3_ glycols could be efficiently produced because Ni can promote the hydrogenation while W promotes bond cleavage via a retro-aldol condensation^8^. Hence, to obtain a more satisfactory C_2_/C_3_ glycols yield, the 7Ni-20W/β (Ni-W/β for short) system was applied in the following study.

### The effect of co-catalysts on product distribution

Although the conversion of cellulose into glycols has been widely studied, controlling the C_2_/C_3_ distribution has received less attention, and the increase of PG yield is hard because rigorously controlling the C-C bond cleavage position is important^6,8–12,14,16^. Hence, to control the position of C-C bond cleavage to increase the selectivity of PG and investigate the influence of these co-catalysts on the C_2_/C_3_ glycols distribution, a series of metal oxides and alkalis were added into the Ni-W/β system, as displayed in Table 1. ZnO, Fe_3_O_4_, MgO and alkalis gave rise to improved yields of C_3_ glycols from 19.3 to 35.8, 24.1, 23.2, 26.8, 29.3 and 32.1%, and the yields of C_2_/C_3_ glycols improved from 64.6 to 70.1 and 65.3% with ZnO and Fe_3_O_4_, respectively. Among these co-catalysts, ZnO significantly influenced the C_2_/C_3_ glycols distribution and PG yield, which was further investigated. First, the effect of the ZnO dosage was tested, as shown in Figure S4. Opposite trends in the PG and EG distribution were observed because the PG yield (C_3_) increased significantly with the addition of ZnO, while the EG (C_2_) yield obviously decreased. The balance between the yields of PG and EG indicated that PG was preferentially obtained in the presence of ZnO, i.e., the selective generation of PG and EG was influenced by ZnO^27^. Furthermore, LA, another C_3_ product, could not be generated when there was no ZnO in the system. In addition, its yield increased with increasing ZnO dosage (less than 200 mg). Therefore, from the increasing trends in the yields of PG (C_3_) and LA (C_3_) and the decreasing trends in the yields of EG (C_2_) and S (C_6_), we can infer that ZnO selectively promoted the formation of C_3_ products via C_3_-C_3_ bond cleavage from C_6_. Since there are few reports about the specific role of ZnO^28,29^, we further investigated the mechanism of the effect of ZnO in the mechanism section.

**Table 1:** The catalytic performance of co-catalysts based on Ni-W/β.

Entry Catalyst		Conversion (%)	Yield (%)					PG/Glycols (%)
			EG	PG	S	M	Glycols	
1	None	74.0	0.0	0.0	0.0	0.0	0.0	0.0
2	Ni-W/β	100.0	45.3	19.3	11.7	3.4	64.6	29.9
3	Ni-W/β + ZnO	100.0	34.3	35.8	6.6	7.3	70.1	51.1
4	Ni-W/β + Fe_3_O_4_	100.0	41.2	24.1	17.0	10.1	65.3	36.9
5	Ni-W/β + MgO	100.0	39.8	23.2	10.5	5.6	63.0	36.8
6	Ni-W/β + Al_2_O_3_	100.0	43.3	10.7	14.7	3.5	54.0	19.8
7	Ni-W/β + TiO_2_	100.0	41.7	12.6	10.8	7.8	54.3	23.2
8	Ni-W/β + NaOH	100.0	23.3	26.8	4.9	4.8	50.1	50.1
9	Ni-W/β + Ca(OH)_2_	100.0	27.8	29.3	6.2	9.8	57.1	51.3
10	Ni-W/β + Ba(OH)_2_	100.0	25.7	32.1	4.1	6.2	57.8	55.5

Reaction conditions: 30 min, 6 MPa H_2_, 245 °C, 50 mL of H_2_O, 0.5 g of cellulose, 0.15 g of catalyst, 100 mg of co-catalysts. EG: ethylene glycol; PG: 1,2-propylene glycol; S: sorbitol; and M: mannitol.

### Reaction mechanism

**Figure 4:**
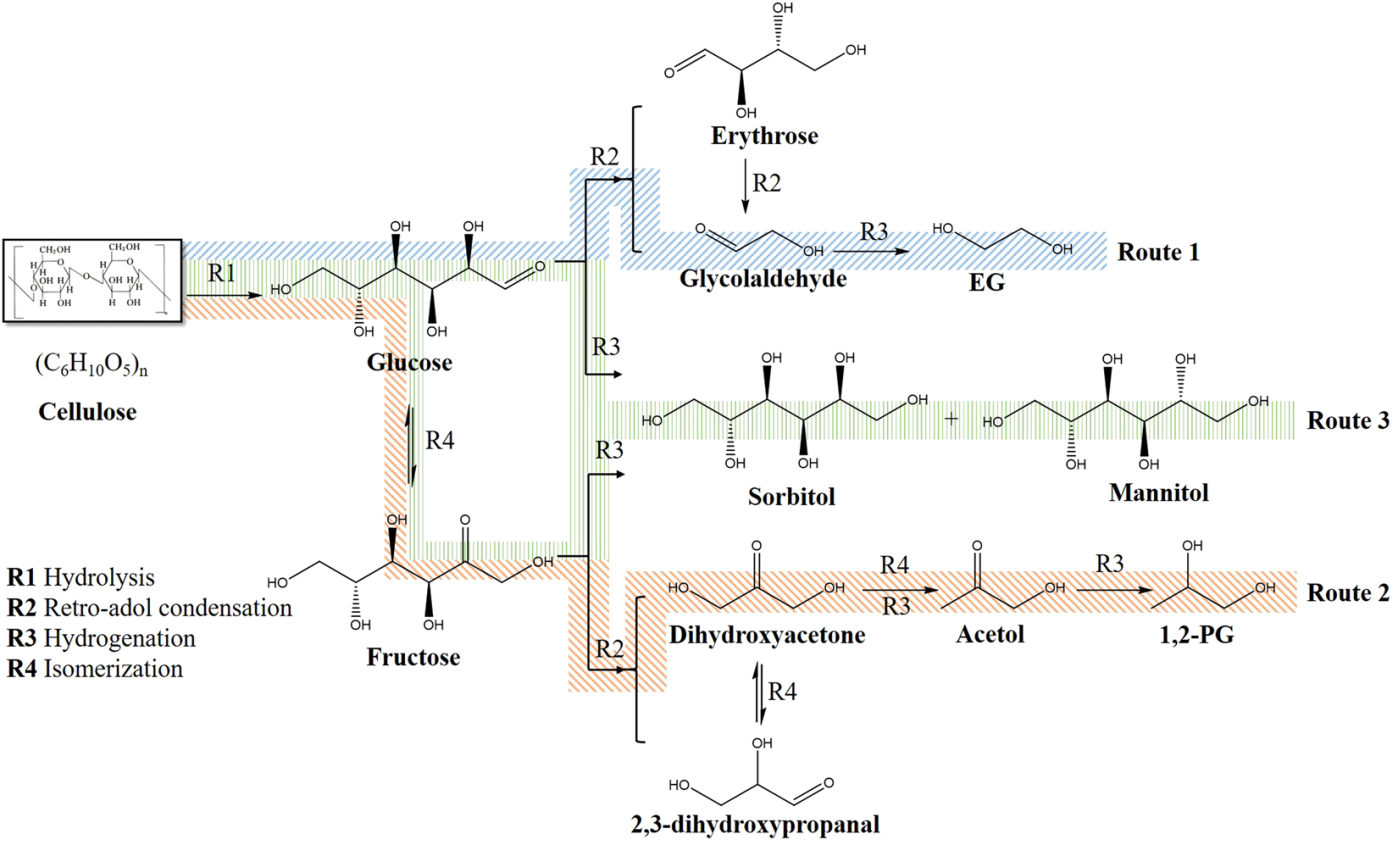
Proposed reaction pathway for the transformation of glucose into propylene glycol on Ni-W/β in the presence of ZnO.

The proposed reaction route for the formation of C_2_/C_3_ glycols from cellulose using Ni-W-ZnO/β is shown in Fig. 4. Based on this reaction route, the final products, EG, PG, sorbitol and mannitol, were produced by route 1 (R1: hydrolysis, R2: retro-aldol condensation and R3: hydrogenation), route 2 (R1: hydrolysis, R4: isomerization, R2, R4/R3 and R3) and route 3 (R1 and R3), respectively. Figure 1 shows that when β-zeolite served as the support, the maximum total yield of the four final products was obtained. We conjectured in support section above that the excellent performance of β-zeolite might be attributed to the enhancement of cellulose hydrolysis. Here, this hypothesis was further supported. As shown in Table S4, when using Ni-W/raw-β as the catalyst, the total yield of the four polyols produced from glucose was 64.9%, while that from cellulose was 61.2%. This result suggested that the pseudo yield of R1 (Fig. 4) was 94.3, which was obviously higher than the conversion efficiencies obtained with other catalysts. Therefore, it could be concluded that the acidity of Ni-W/raw-β enhanced cellulose hydrolysis. Furthermore, as the strength of the acidity was enhanced by dealumination, Ni-W/β provided a higher pseudo yield of the hydrolysis than that obtained with Ni-W/raw-β.

The proposed reaction route (Fig. 4) was also utilized to explain the synergistic effects of Ni and W. As shown in Fig. 3, a significant difference in the product distribution when using different Ni-W loadings was observed. From the perspective of W, when the loading of W was insufficient (e.g., 7Ni-5W), the yields of sorbitol and mannitol (C_6_ products) were significantly higher than that of C_2_/C_3_ glycols, suggesting that route 3 (Fig. 4) was favoured over routes 1 and 2. When the W loading was sufficient (e.g., 7Ni-15W), routes 1 and 2 were favoured over route 3. The significant difference between the results with insufficient and sufficient loadings of W indicated that W promoted routes 1 and 2 in which bond cleavage from C_6_ into C_2_/C_3_ (R2 retro-aldol) was the key step. From the perspective of Ni, the yields of the four major polyol products all decreased when the Ni loading was insufficient (e.g., 1Ni-20W), while a high yield of polyols was observed when sufficient Ni was present (e.g., 7Ni-20W). This might be because hydrogenation (R3), as the common formation step of these four products in routes 1, 2 and 3, was disfavoured when Ni was insufficient. In other words, Ni facilitated the hydrogenation step. The fact that Ni facilitated the hydrogenation step could also explain why the yields of sorbitol and mannitol were significantly higher than those of C_2_/C_3_ glycols when the loading of Ni was higher (e.g., 15Ni-20W, Fig. 3). Excess Ni promoted route 3 because sorbitol and mannitol were readily generated only though glucose hydrogenation^30,31^. In summary, W worked on R2, while Ni acted on R3, and the favourability of routes 1, 2 or 3 was determined by the synergy of Ni and W. Based on the above mechanism, 7Ni-20W/β successfully drove the reaction through routes 1 and 2 and ultimately provided the maximum yield of C_2_/C_3_ glycols.

To further understand the mechanism of the hydrogenolysis of cellulose into C_2_/C_3_ products, the reactions of the possible intermediates (such as glucose, fructose, sorbitol, mannitol, dihydroxyacetone and acetol) were performed, as shown in Table 2. As displayed in Entry 1, sorbitol and mannitol were stable and did not readily undergo further conversion^27,32,33^, while glucose and fructose (Table 2, entries 3 and 5) were readily converted to EG and PG through hydrogenolysis, indicating that if the target products were C_2_/C_3_, R2 should occur more quickly than R3. This result also supported the above discussion that controlling the synergy of the hydrogenation (Ni) and bond cleavage (W) was important. Furthermore, the reactions of glucose, fructose, dihydroxyacetone and acetol were performed in the presence and absence of ZnO to understand how ZnO influences the hydrogenolysis of cellulose into C_3_ products. As shown in entries 3 to 10, in the absence of ZnO (Entry 3), glucose was converted to EG in a yield of 51% and PG in 14.5%, suggesting that route 1 is favoured over route 2 (Fig. 4). When ZnO was present (Entry 4), glucose was converted to EG in a yield of 28.9% and PG in 35.7%, suggesting that route 2 is favoured over route 1. The results under different ZnO conditions indicated that ZnO could promote route 2, suggesting ZnO influences a specific step. The reaction results of fructose, dihydroxyacetone and acetol in the presence and absence of ZnO (Entries 5–10) showed that ZnO had no impact on the conversion of fructose to 1,2-PG, dihydroxyacetone to 1,2-PG and acetol to 1,2-PG. As Fig. 4 shows, excluding the above steps, we could conclude that R4 (the isomerization of glucose to fructose) was the major step influenced by ZnO. In addition, the results of dihydroxyacetone (Entries 7 and 8) further demonstrated that ZnO had no significant effect on the isomerization of dihydroxyacetone, indicating that the effect of ZnO on isomerization in this reaction system was specific to the promotion of glucose isomerization.

**Table 2:** Conversion of different carbohydrates as probe reactants over Ni-W/β.

Entry	Catalyst	Reactant	Yield (%)			
			EG	PG	S	M
1	Ni-W/β	Sorbitol	0.0	0.0	95.0	0.0
2	Ni-W/β	Mannose	12.3	8.9	1.3	51.5
3	Ni-W/β	Glucose	51.0	14.5	8.8	1.7
4	Ni-W/β + ZnO	Glucose	28.9	35.7	7.2	2.3
5	Ni-W/β	Fructose	13.2	48.7	10.2	8.9
6	Ni-W/β + ZnO	Fructose	12.9	49.2	9.9	9.5
7	Ni-W/β	Dihydroxyacetone	0.0	69.8	—	—
8	Ni-W/β + ZnO	Dihydroxyacetone	0.0	71.2	—	—
9	Ni-W/β	Acetol	0.0	88.7	—	—
10	Ni-W/β + ZnO	Acetol	0.0	87.4	—	—

Reaction conditions: 30 min, 6 MPa H_2_, 245 °C, 50 mL of H_2_O, 0.5 g of reactant, 0.15 g of catalyst, and 100 mg of ZnO. EG: ethylene glycol; PG: 1,2-propylene glycol; S: sorbitol; and M: mannitol.

### Reusability

**Figure 5:**
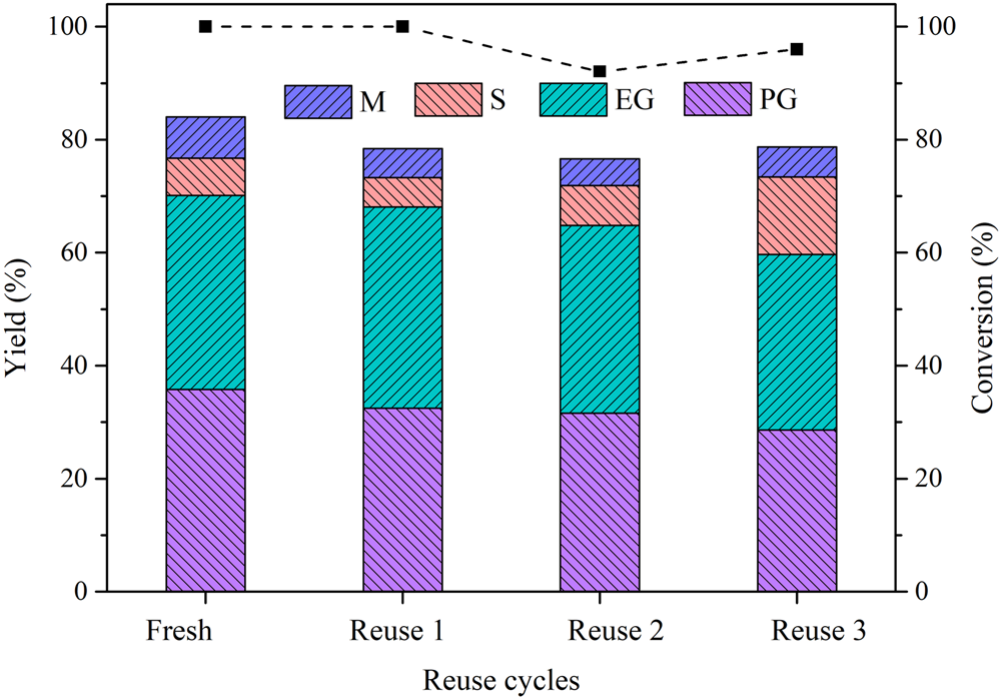
Reuse of the ZnO and Ni-W/β catalyst system. Reaction conditions: 30 min, 6 Mpa H_2_ 245 °C, 50 mL of H_2_O, 0.5 g of cellulose, 0.15 g of catalyst, and 100 mg of ZnO. EG: ethylene glycol; PG: 1,2-propylene glycol; S: sorbitol; and M: mannitol.

Catalyst recycling is an important property for the practical application of catalysts in metal-catalysed liquid-phase reactions for the following two major reasons: 1) the catalyst can readily leach metal, which is attributed to the H^+^ formed in water at high temperature^34,35^, and 2) the complex intermediate components produced by the side reactions can block the pores and poison the catalyst^36,37^. Therefore, the reusability of Ni-W-ZnO/β and the changes in its composition and structure were tested, and the results are displayed in Fig. 5, Table S2 and Table 3. As Fig. 5 shows, the yield of total glycols decreased from 70.1% to 68.1%, 64.8% and 59.7% by the second, third and fourth runs, respectively, while the conversion of cellulose remained at 100%, 96% and 92%. The slight decrease could be attributed to structural changes, metal leaching from the catalyst and acidity decrease, which was verified by N_2_-adsorption and desorption (Table S2) and ICP results (Table 3) and NH_3_-TPD (Table S3), respectively. As shown by Table S2, entries 9 and 15–17, with the reuse of the catalyst, the BET surface area of Ni-W/β decreased slightly from 287 to 255 m^2^/g. It could be speculated that deposition of carbon species and collapse of the mesopores occurred during reuse, changing the catalytic structure^38^. Nevertheless, differences between the pore volume and size in fresh and used catalysts were not significant, suggesting that the catalytic structure was not badly damaged by four cycles of reuse^39^. As displayed in Table 3, the loading of Ni decreased from 7.29 to 6.41, 5.73, 5.64 and 5.58 wt% over four cycles of reuse, while W decreased from 20.87 to 18.11, 15.26, 13.58 and 11.56 wt%. As shown in Table S3 (Entries 4, 10–12), the acidity of the catalyst decreased during four cycles reaction, which may attributed to the leaching of Ni and W and the decreased BET surface area. What’s more, the increasing tendency of Ni/W weight ratio (from 0.35 to 0.41) suggested that the leaching W was faster than Ni, indicating the reaction route might be changed by the synergy of Ni and W. In the first and second cycles (Fresh and Reuse 1), the loadings of Ni and W were relatively balance. In this case, the high C_2_/C_3_ glycol yields (70.1% and 68.1%) could be attributed to the balance loading of Ni and W, and the slight decrease might be caused by the leaching of Ni and W. In the third and fourth cycles (Reuse 2 and 3), the yield of C_2_/C_3_ glycols decreased to 64.8% and 59.7%, respectively, while the yields of mannitol and sorbitol were significantly increased. According to the above mechanism (Fig. 4), W promoted R2, while Ni promoted R3, and the favourability of routes 1, 2 or 3 was determined by the synergy of Ni and W. Take the fourth cycle (Reuse 3) as an example, the loading of W was insufficient because of the faster leaching of W than Ni. Therefore, R2 in routes 1 and 2 was disfavoured, while R3 in route 3 was favoured, which led to an increase in the mannitol and sorbitol yield and a decrease in EG and 1,2-PG. According to the above results and discussion, the loading of Ni and W and the balance of hydrogenation and RAC are also greatly important during reuse. What’s more, because of the leaching of Ni and W was obvious and could not be neglected, the effect of leached Ni and W on cellulose hydrogenolysis into PG/EG was investigated (Table S5). The results suggested that when the supported W was sufficient, the leached W did not work significantly on PG/EG production, while there was no supported W, the leached W could play a role in the reaction, which was the evidence that supported W, other than leached W, had a major role on PG/EG production in our catalytic system.

**Table 3:** Changes in the nickel, tungsten and zinc contents in the 7Ni-20W/β + ZnO system during four cycles of reuse.

Cycle	Ni (wt %)		Ni balance (wt %)^c^	W (wt %)		W balance (wt %)^c^	Zinc (%)		Zn balance (%)^c^	Ni/W weight ratio
	Liquid^a^	Solid^b^		Liquid	Solid		Liquid	Solid		
Fresh	—	7.29	—	—	20.87	—	—	100.00	—	0.35
Reuse 1	1.08	6.41	+ 0.20	2.62	18.11	−0.14	3.52	96.70	+ 0.22	0.35
Reuse 2	0.62	5.73	−0.06	3.01	15.26	+ 0.16	4.19	91.92	−0.59	0.38
Reuse 3	0.34	5.64	+ 0.25	1.79	13.58	+ 0.11	2.99	88.65	−0.28	0.41
after	0.21	5.58	+ 0.15	2.03	11.56	+ 0.01	3.16	85.76	+0.27	0.48

^a^Liquid (wt% or %) = the weight of Ni, W or Zn in the liquid products after the reaction. The weight of Ni and W in the liquid products was converted into equivalent loadings in the catalyst (wt %).

^b^Solid (wt% or %) = the weight of Ni, W or Zn in the solid catalysts after the reaction.

^c^Ni, W or Zn balance (wt% or %) = the weight of Ni, W or Zn in the liquid products + the weight of Ni, W or Zn in the solid catalysts – the weight of Ni, W or Zn before the reaction.

## Conclusion

To promote the selective transformation of cellulose into C_2_/C_3_ glycols and increase the proportion of C_3_ glycol in the glycol products, we attempted to control the synergy of the retro-aldol condensation, hydrogenation and isomerization reactions. After the optimization of the catalyst support, the Ni-W loading and the co-catalyst, ZnO-modified 7Ni-20W/β was employed, and the influence of these factors on the product distribution was discussed. Compared to supports such as Al_2_O_3_, TiO_2_, SiC and ZSM-5, raw β-zeolite exhibited the best catalytic performance, which was attributed to its ability to promote the hydrolysis of cellulose into small molecules by its abundant acid sites. The synergy of Ni and W was the key to the reaction: on the one hand, the synergy of Ni and W could provide both Lewis and Brønsted acid sites in the catalyst and sequentially promote the formation of C_2_/C_3_ glycols; on the other hand, Ni is effective in the hydrogenation, and W facilitates the bond cleavage (C_6_ to C_2_/C_3_). The optimum loadings of Ni and W were 7 wt% and 20 wt% because an unbalanced loading of Ni and W had a detrimental influence on the synergy of the hydrogenation and retro-aldol condensation. Moreover, with the leaching of the metals during four cycles of reuse, the catalytic performance was also influenced by the synergy of Ni and W primarily through the change in their loading. In addition, by screening a series of metal oxides and alkalis as co-catalysts in the Ni-W/β system, ZnO caused the greatest improvement in the C_2_/C_3_ yield. The increased yield of C_3_ glycol by ZnO was attributed to the promotion of the isomerization of glucose to fructose. After the systematic optimization of the catalyst support, the Ni-W loading and the co-catalyst, the yield of C_2_/C_3_ glycols reached 70.1%, with propylene glycol accounting for 51.1% of the product, under 4 MPa hydrogen pressure at 245 °C for 30 min.

## Methods

### Catalyst Preparation

Commercial H-β-zeolite (Catalyst Plant of Nankai University), denoted as “raw β”, was firstly dealuminated by refluxing in nitric acid at 80 °C at a solid-to-liquid ratio of 1 g: 20 mL under a stirring speed of 200 r/min for 20 h^25^. After dealumination, denoted as “β”, the zeolite powder was collected by centrifugation and then washed with deionized water several times until the supernatant was neutral. Finally, the obtained β-zeolite was dried at 150 °C overnight.

The catalysts, including Ni-W/raw-β, Ni-W/β, Ni-W/Al_2_O_3_, Ni-W/SiC, Ni-W/ZSM-5 and Ni-W/TiO_2_, were prepared by incipient wetness impregnation, dried at 120 °C overnight, calcined at 500 °C for 4 h, and reduced under a hydrogen-nitrogen flow (volume ratio = 5%: 95%) at 500 °C for 4 h. In detail, the Ni-W/β catalysts were prepared by co-impregnation with an aqueous solution of ammonium metatungstate hydrate (AMT) and Ni(NO_3_)_2_·6H_2_O. Regarding the nickel and tungsten loadings, the catalyst with 25 wt % of tungsten and 5 wt % of the nickel, for example, is denoted 5Ni–25 W/β. For all of the above catalysts, the nickel and tungsten loadings were 7 wt % and 20 wt %, respectively. All chemicals were purchased from Sinopharm Chemical Reagent Co. Ltd.

### Analytical methods

The specific surface area, pore volume and pore diameter of the catalysts were tested by N_2_ adsorption–desorption (Micromeritics ASAP 2020)^40^. Each sample was purged in a vacuum at 300 °C for 3 h before analysis. The Brunauer-Emmett-Teller (BET) method was used to calculate the specific surface areas of the catalysts, the pore volume was calculated by the Barrett-Joyner-Halenda (BJH) model and the pore diameter was calculated by the BJH method from the desorption branches. Powder X-ray diffraction (XRD) patterns were collected on an X-ray powder diffractometer (Bruker D8 Advance) with Cu Kα radiation (40 kV, 40 mA) over a 2θ range of 5° to 80° at room temperature. Transmission electron microscopy (TEM) images were recorded on a JEOLJEM-1230 instrument operated at 80 kV.

The acidic properties of the catalysts were tested by NH_3_ temperature-programmed desorption (NH_3_-TPD) and pyridine infrared (Py-IR) spectroscopy^20,40^. The NH_3_-TPD was conducted using a Micromeritics AutoChemII 2920 system. The sample was pretreated in helium at 300 °C for 60 min, after which the sample was cooled to 120 °C in a helium flow. Next, 5% NH_3_ in the helium flow was absorbed onto the sample at 120 °C for 120 min. Finally, NH_3_-TPD was carried out from 120 °C to 800 °C at a ramp rate of 10 °C/min in helium flow^20^. The Py-IR spectra were recorded on a Perkin Elmer Frontier FT-IR in the range of 1400–1700 cm^−1^ with a spectral resolution of 2 cm^−1^. A 10-mg sample of catalyst was pressed into a wafer with a diameter of 13 mm and then set in a quartz IR cell. The catalysts were dried at 400 °C for 2 h under vacuum. After cooling, pyridine vapor was injected into the cell, and the adsorption period lasted for 30 min. Subsequently, the desorption profiles at 150 °C (1 h), 250 °C (1 h), 350 °C (1 h), and 400 °C (1 h) were recorded^40,41^.

The confirmation of thorough dealumination in β, the metal loadings in the solid catalyst and the leaching of the metals into the liquid were determined by inductively coupled plasma optical emission spectroscopy (ICP-OES, Perkin Elmer Optima 2100 DV). Prior to the measurements, the solid samples were digested in an acidic mixture (HF:HNO_3_:HClO = 1:1:1), and the liquid samples were digested in HNO_3_ at 150 °C for 12 h.

### Catalytic Reaction

The catalytic experiments were carried out in a 100-mL stainless steel autoclave with a Teflon insert, and the operating parameters were the same as those in our previous study^40^. To the autoclave were added 0.5 g of cellulose, 150 mg of catalyst, and 50 mL of water. Microcrystalline cellulose (Shanghai Chineway Pharm. Tech. Co. Ltd) was dried under vacuum at 105 °C for 12 h before use. After the reactor was sealed, the vessel was purged with nitrogen three times to exclude air and then pressurized with 6 MPa of hydrogen and heated to the desired reaction temperature, which was kept constant throughout the reaction with stirring at 1000 rpm. The zero point of the “reaction time” was defined as the time point at which the required temperature was reached. When the reaction ended, the reactor was immediately quenched to room temperature in an ice-water bath. The products were quantified by gas chromatography (GC, Agilent 7820 A, J&W125–7332, 30 m × 530 μm × 1 μm) with an FID detector and high-performance liquid chromatography (HPLC, Agilent 1200, Shodex SUGAR SH1011) with VWD and RI detectors. The cellulose conversion (%) was determined from the change in the cellulose weight from before to after the reaction, and this value was further verified by measuring the total organic carbon (TOC-VCPH, Shimadzu, Japan). The yield of products (%) was defined as ${\rm{Yield}}\,( \% )=\frac{\,the\,weight\,of\,carbon\,in\,one\,product\,after\,reaction}{the\,weight\,of\,carbon\,in\,cellulose\,before\,reaction}\times 100 \%$. For the catalyst reuse studies, the solid catalysts were collected by filtration and washed several times with water. The recovered catalysts were used after reduction in a mixed hydrogen-nitrogen flow (volume ratio = 5%: 95%) at 280 °C for 4 h to remove residual cellulose^42^ and keep Pt in its metallic state.

## Supplementary information

Supplementary figures and tables
