# Supplementary material for: Reaction Route Selection for Cellulose Hydrogenolysis into C2/C3 Glycols by ZnO-Modified Ni-W/β-zeolite Catalysts
Source: Sci Rep. 2019 Aug 16;9:11938. doi: 10.1038/s41598-019-48103-6 (PMC6697703; doi:10.1038/s41598-019-48103-6)
Supplement: Supplementary file 1 — Supplementary figures and tables [file 41598_2019_48103_MOESM1_ESM.pdf]

Supplementary Information for

Reaction Route Selection for Cellulose Hydrogenolysis into C_2/C_3 Glycols by ZnO-Modified Ni-W/β-zeolite Catalysts

Minyan Gu [1], Zheng Shen [1,*], Long Yang [1], Wenjie Dong [2], Ling Kong [1], Wei Zhang [1], Boyu Peng [1], and Yalei Zhang [1,*]

[1] State Key Laboratory of Pollution Control and Resources Reuse, Key Laboratory of Yangtze River Water Environment of MOE, Tongji University, Shanhai, 200092, China

[2] College of Quality and Safety Engineering, China Jiliang University, Hangzhou, 310018, China

* E-mail: 78shenzheng@tongji.edu.cn, zhangyalei@tongji.edu.cn, Tel/Fax: +86 21 65985811.

Figures S1-S4

Tables S1-S5

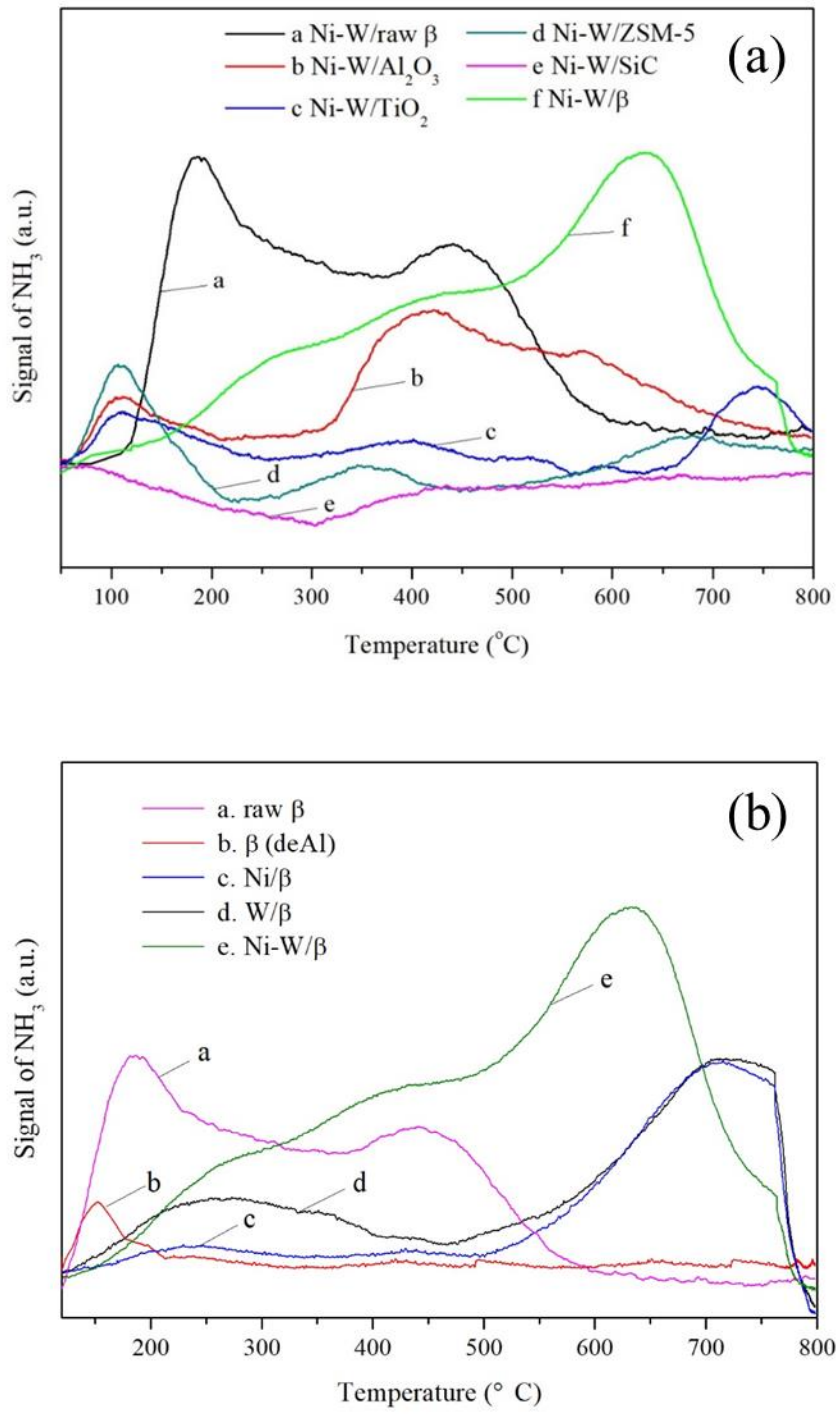

Figure S1. NH_3-TPD curves of catalysts.

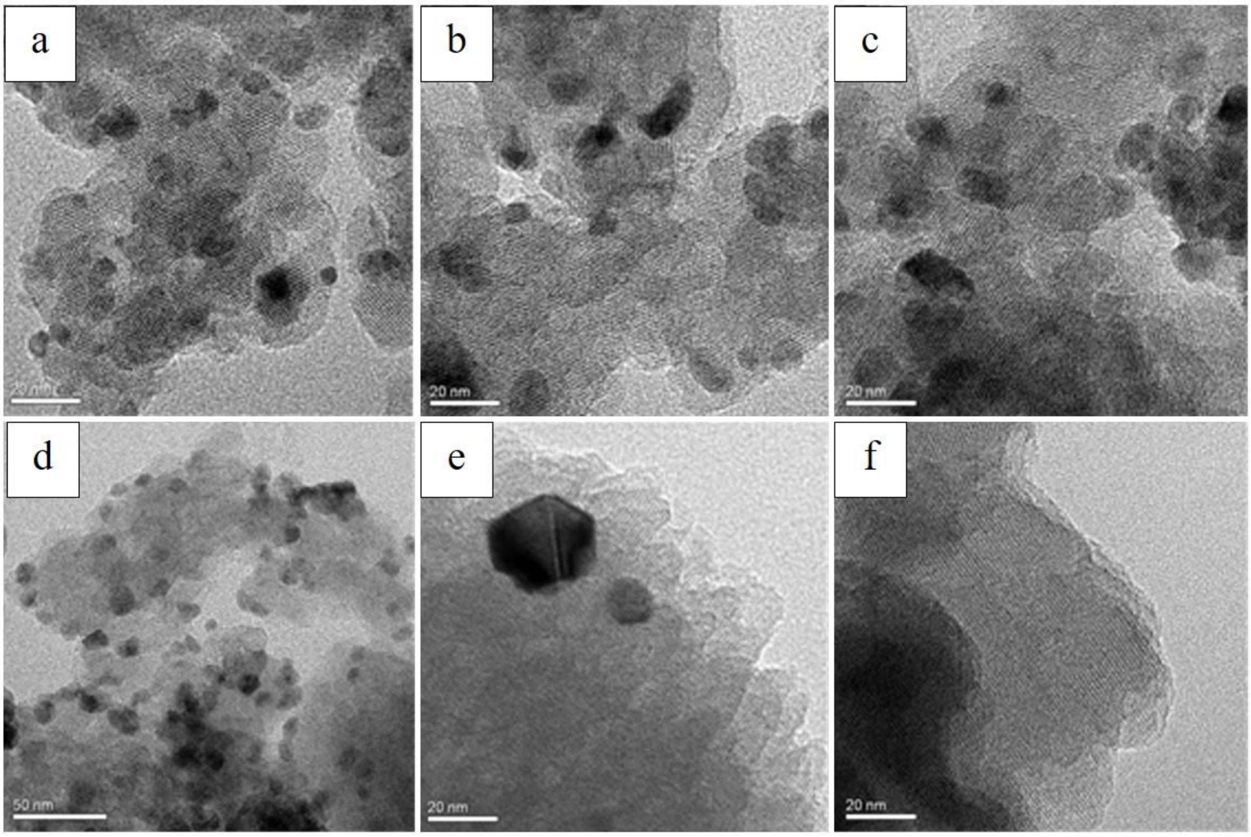

Figure S2. Transmission electron microscope (TEM) of Ni-W/β catalysts: a) 1Ni-20W/β; b) 3Ni-20W/β; c) 5Ni-20W/β; d) 7Ni-20W/β; e) 15Ni-20W/β; f) β-zeolite.

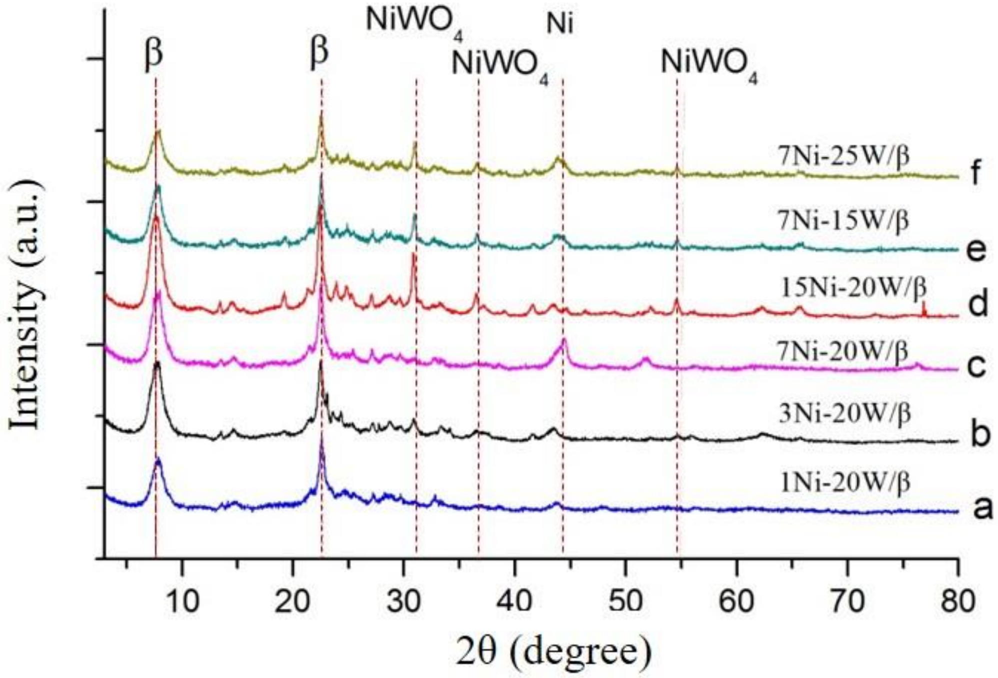

Figure S3. Powder X-ray diffraction patterns (XRD) of Ni-W/β catalysts.

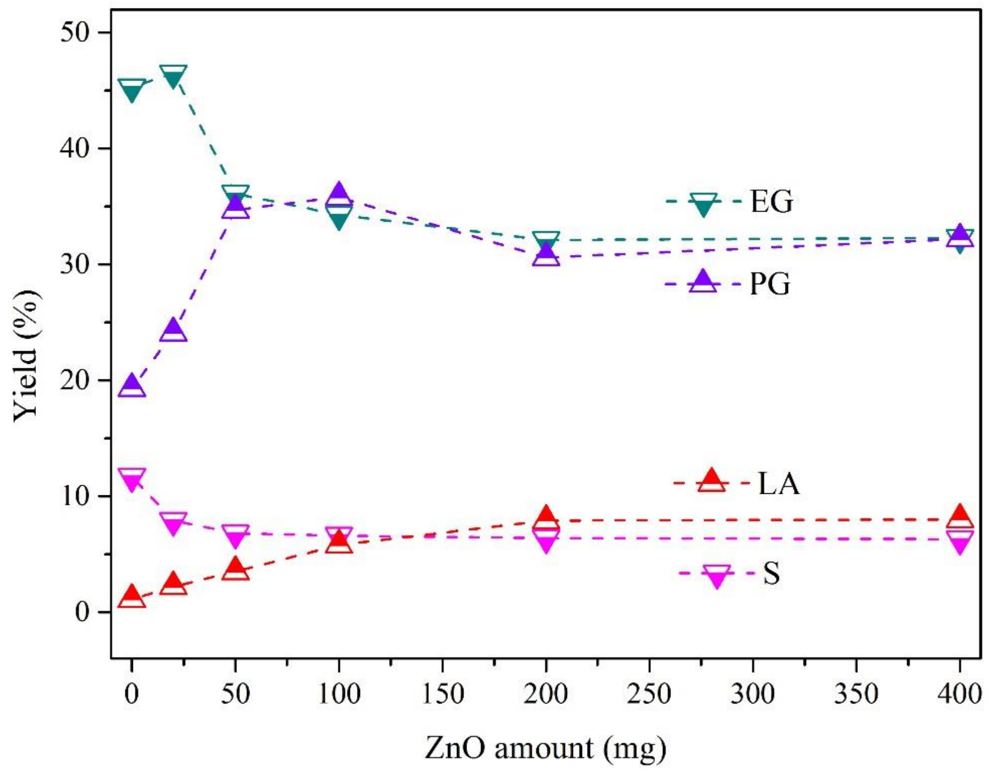

Figure S4. Effect of amount of ZnO combined with Ni-W/β catalyst on the transformation of cellulose into glycols.

Reaction condition: 30 min, 6 MPa H_2, 245°C, 50 mL H_2O, 0.5 g cellulose, 0.15 g catalyst, 0-400 mg ZnO. EG: Ethylene glycol; PG: 1,2-propylene glycol; S: Sorbitol; LA: Lactic acid.

Table S1. Physicochemical properties of the catalysts.

Entry	Catalyst	Metal loading (wt %)		BET surface area (m^2/g)	Acidity (mmol/g)	Yield (%)	
		Ni	W			EG+PG	EG+PG+M+S
1	Ni-W/SiC	7.06	20.70	56	--	32.1	37.9
2	Ni-W/TiO_2	7.21	20.91	42	0.15	38.2	40.5
3	Ni-W/ZSM-5	7.24	21.02	410	0.17	32.8	45.6
4	Ni-W/Al_2O_3	7.14	20.72	134	0.62	29.6	50.1
5	Ni-W/raw-β	7.01	20.55	317	1.71	42.2	61.2
6	Ni-W/β	7.29	20.87	329	1.67	64.6	79.7
7	Raw β	-	-	397	1.03	-	-
8	β(deAl)	-	-	375	0.02	-	-
9	Ni/β	7.12	-	331	0.57	9.5	55.7
10	W/β	-	20.16	345	0.63	4.1	4.1

Table S2. The N_2 adsorption and desorption results of catalysts

Entry	Catalyst	BET Surface Area(m^2/g)	Pore Volume(cm^3/g)	Pore Size (nm)
1	Ni-W/SiC	56	0.10	6.07
2	Ni-W/TiO_2	42	0.09	3.76
3	Ni-W/ZSM-5	410	0.39	3.45
4	Ni-W/Al_2O_3	134	0.31	3.90
5	Ni-W/raw-β	317	0.31	3.92
6	1Ni-20W/β	368	0.35	4.02
7	3Ni-20W/β	357	0.34	4.01
8	5Ni-20W/β	342	0.32	3.99
9	7Ni-20W/β	329	0.32	4.05
10	9Ni-20W/β	313	0.31	4.02
11	15Ni-20W/β	287	0.28	4.07
12	7Ni-5W/β	371	0.36	3.98
13	7Ni-15W/β	348	0.32	4.02
14	7Ni-25W/β	277	0.27	4.05
15	15Ni-20W/β The second run	272	0.27	3.97
16	15Ni-20W/β The third run	268	0.26	3.96
17	15Ni-20W/β The fourth run	255	0.26	3.85

Table S3. The acidity of different catalysts tested by NH_3-TPD

Entry	Catalyst	Acidity (mmol/g)
1	1Ni-20W/β	1.24
2	3Ni-20W/β	1.38
3	5Ni-20W/β	1.68
4	7Ni-20W/β	1.67
5	9Ni-20W/β	1.23
6	15Ni-20W/β	0.52
7	7Ni-5W/β	1.16
8	7Ni-15W/β	1.44
9	7Ni-25W/β	0.97
10	7Ni-20W/β (Reuse 1)	1.58
11	7Ni-20W/β (Reuse 2)	1.33
12	7Ni-20W/β (Reuse 3)	1.25

Table S4. Conversion of cellulose and glucose as the probe reactants over different catalysts.

Catalyst	Reactant	Yield (%) [a]				$Sum_{cellulose}$ or $Sum_{glucose}$ (%)	The pseudo-yield of cellulose hydrolysis [b]
		PG	EG	S	M		
Ni-W/SiC	cellulose	5.2	26.9	4.7	1.1	37.9	65.0
Ni-W/SiC	glucose	6.8	31.1	10.6	9.8	58.3	
Ni-W/TiO2	cellulose	1.1	37.1	1.7	0.6	40.5	73.9
Ni-W/TiO2	glucose	6.9	31.3	9.7	6.9	54.8	
Ni-W/ZSM-5	cellulose	2.1	30.7	9.1	3.7	45.6	66.5
Ni-W/ZSM-5	glucose	11.5	34.7	15.9	6.5	68.6	
Ni-W/Al_2O_3	cellulose	6.8	22.8	15.5	5.0	50.1	81.6
Ni-W/Al_2O_3	glucose	7.6	27.5	19.7	6.6	61.4	
Ni-W/raw-β	cellulose	11.5	30.7	16.6	2.4	61.2	94.3
Ni-W/raw-β	glucose	9.9	39.2	12.7	3.1	64.9	
Ni-W/β	cellulose	19.3	45.3	11.7	3.4	64.6	98.6
Ni-W/β	glucose	51.0	14.5	8.8	1.7	65.5	

[a] Reaction condition: 30 min, 6 MPa H_2, 245°C, 50 mL H_2O, 0.5 g reactant, 0.15 g catalyst.

EG: Ethylene glycol; PG: 1,2-propylene glycol; S: Sorbitol; M: Mannitol.

[b] The pseudo-yield of cellulose hydrolysis= (the sum yield of PG, EG, S an M from cellulose)/(the sum yield of PG, EG, S an M from cellulose)

Table S5. The catalytic performance of cellulose hydrogenolysis into EG/PG via different catalytic conditions

Entry	Catalyst	Yield (%) [b]			
		PG	EG	S	M
1	None	0	0	0	0
2	Solution [a]	0	0	0	0
3	Solution [a]	0	0	0	0
4	Solution [a]	0	0	0	0
5	W/β	0	4.1	0	0
6	W/β+Solution	0	1.1	0	0
7	W/β+Solution	0	0	0	0
8	W/β+Solution	0	2.7	0	0
9	Ni/β	2.6	6.9	44.6	11.1
10	Ni/β+Solution	3.8	9.2	34.7	12.3
11	Ni/β+Solution	3.6	8.4	39.6	12.6
12	Ni/β+Solution	4.7	9.9	37.2	9.6
13	Ni-W/β	19.3	45.3	11.7	3.4
14	Ni-W/β+Solution	18.9	42.6	12.8	5.6
15	Ni-W/β+Solution	20.6	45.9	10.9	3.1
16	Ni-W/β+Solution	18.7	44.7	12.1	4.9

[a] Solution (contains leached Ni and W) was prepared by reaction condition (30 min, 6 MPa H_2, 245°C, 50 mL of H_2O, 0.15 g of Ni-W/β)

[b] Reaction condition: 30 min, 6 MPa H_2, 245°C, 50 mL of H_2O, 0.5 g of cellulose, 0.15 g of catalyst
